# Combination coating of chitosan and anti-CD34 antibody applied on sirolimus-eluting stents can promote endothelialization while reducing neointimal formation

**DOI:** 10.1186/1471-2261-12-96

**Published:** 2012-10-26

**Authors:** Feng Yang, Shi-Chao Feng, Xiang-Jun Pang, Wei-Xiao Li, Yong-Hua Bi, Qian Zhao, Shi-Xuan Zhang, Yang Wang, Bo Feng

**Affiliations:** 1 Interventional Radiology Department, The First Affiliated Hospital of China Medical University, 155 Nanjing North Street, Shenyang 110001, Liaoning, PR China; 2 Key Laboratory of Diagnostic Imaging and Interventional Radiology, 5 Nanqi Western Road, Shenyang, 110024, Liaoning Province, PR China; 3 Fei Cheng Hospital of Traditional Chinese Medicine, 024 Chang Shan Street, Feicheng, 271601, Shandong, PR China; 4 Department of Laboratory Animals, General Hospital of Shenyang Military Area Command, No.83, Wenhua Road, Shenhe District, Shenyang, 110840, PR China; 5 Dalian University of Technology, No.2 Linggong Road, Ganjingzi District, Dalian City, 116024, Liaoning, PR China

**Keywords:** Anti-CD34 antibody, Endothelial progenitor cells, Hyaluronan and chitosan coating, Scanning electron microscopy

## Abstract

**Background:**

Circulating endothelial progenitor cells (EPCs) capture technology improves endothelialization rates of sirolimus-eluting stents (SES), but the problem of delayed re-endothelialization, as well as endothelial dysfunction, has still not been overcome. Therefore, we investigated whether the combination coating of hyaluronan-chitosan (HC) and anti-CD34 antibody applied on an SES (HCASES) can promote endothelialization, while reducing neointimal formation and inflammation.

**Methods:**

Sirolimus-eluting stents(SES), anti-CD34 antibody stents (GS) and HC-anti-CD34 antibody combined with sirolimus-eluting stents (HCASES) were deployed in 54 normal porcine arteries and harvested for scanning electron microscopy (SEM) and histological analysis. The ratio of endothelial coverage above the stents was evaluated by SEM analysis at 7, 14 and 28 days. The percentage of in-stent stenosis was histologically analyzed at 14 and 28 days.

**Results:**

SEM analysis at 7 days showed that endothelial strut coverage was increased in the HCASES group (68±7%) compared with that in the SES group (31±4%, p=0.02). At 14 days, stent surface endothelialization, evaluated by SEM, showed a significantly higher extent of endothelial coverage above struts in the GS (95 ± 2%) and the HCASES groups (87±4%) compared with that in the SES group (51±6%, p=0.02). Histological examination showed that the percentage of stenosis in the HCASES group was not significantly different to that of the SES and GS groups (both p> 0.05). At 28 days, there was no difference in the rates of endothelial coverage between the HCASES and GS groups. The HCASES group showed less stenosis than that in the GS group (P < 0.05), but it was not significantly different from the SES group (P=0.068).

**Conclusions:**

SEM and histology demonstrated that HCASESs can promote re-endothelialization while enhancing antiproliferative effects.

## Background

The widespread use of drug-eluting stents, allowing programmable localized elution of drugs to inhibit neointimal formation, has considerably reduced the incidence of in-stent restenosis compared with bare metal stents [1-3]. However, the beneficial effect of drug elution is overshadowed by late in-stent thrombosis (LST), caused by delayed re-endothelialization as well as local hypersensitivity reactions potentially related to the drug, the polymer, or both, and this is a potentially fatal complication [4-6]. There is accumulating evidence that restoration of a newly established and functional endothelium is a prerequisite for the effective inhibition of neointimal hyperplasia and stent thrombosis in the vascular repair response [7,8].

The capture of circulating endothelial progenitor cells (EPCs) to an anti-CD34 antibody stent(GS) surface, using an immobilized antihuman CD34 antibody, has been proposed to contribute to accelerate re-endothelialization and decrease thrombogenicity [9]. Moreover, the combination of EPC-capture and drug-elution technology, such as sirolimus-eluting stents (SES) with immobilized GS (SES–anti-CD34 stent), has been shown to enhance the degree of endothelial cell coverage compared with an SES alone [10]. While EPCs improve the percentage of stent strut endothelialization of SESs, endothelial dysfunction is still present, and its long-term consequences remain to be determined, as demonstrated by the occurrence of LST, even with GS [11].

Recently, a novel coating with a “prohealing” approach, hyaluronan -chitosan (HC) multilayer coating, was discovered, which can promote the adhesion, proliferation and differentiation of EPCs. This coating has good biocompatibility as well as anticoagulant activity, which may contribute to the restoration of functional endothelium [12]. In the current study, we tested the hypothesis that HC-anti-CD34 antibody combined with sirolimus-eluting stents (HCASES) enhance the degree of endothelial cell coverage compared with SESs alone.

## Methods

### Devices used in the study

**Figure 1:**
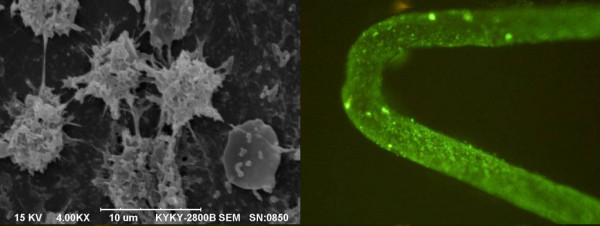
**After implanting CD34 coated stents in swine vessel, EPCs in the peripheral blood can be quickly and specificity captured by CD34 monoclonal antibody on the stent surface,differentiated into vascular endothelial cells by fluorescence immunohistochemistry. **48 hours later,about 85% of stent surface was covered with monolayer vascular endothelial cells by SEM.

The current study used the anti-CD34 antibody stent (GS), the sirolimus-eluting stent (SES), and the HCASES. The control stents included the GS and SES.They are commercially available stents (SES; Cypher, Cordis, Miami, FL, USA, and GS; Genous, OrbusNeich Medical, Fort Lauderdale, FL, USA). Electrostatic self-assembly multilayer-coating endovascular stents loaded with CD34 antibody were prepared (patent WO2009/04955A1). Observing the surface of basis coating by atomic mechanics microscope,there was uniformly dense island arrangement. The bioactive matrix coatings effectively improved hemocompatibility of the metal stent surface by platelet adhesion experiment by scanning electron microscopy. The feasibility of accelerate endothelialization was evaluated by scanning electron microscopy and immunofluorescence (Figure 1). The antibody stents can capture EPCs rapidly in fresh human peripheral blood in vitro. The amount of CD34 antibody loaded per 316 L stainless steel coronary artery stent was 50 ± 18 ng and 8.5± 1.5 μg of sirolimus was loaded for the HCASES. HCASESs were immersed into fresh blood with heparin, incubated at 37°C for 1 h, washed with Tween 80-PBS. The biological activity of the antibody was unchanged after the HCASES was stored at 4°C for half a year.

### Experimental design

The study protocol was approved by the Animal Care and Use Committee of the China Medical University. The present study was performed in accordance with the Guide for the Care and Use of Laboratory Animals (National Research Council, NIH Publication No. 85–23, revised 1996).

In our study, investigators were blinded to the type of stent, which was randomized and deployed in normal coronary arteries in 27 swine (54 coronary arteries) and harvested at 7, 14 and 28 days. Nine swine were sacrificed at each time point (18 stents: six SESs;six Gs;six HCASESs), and they were investigated for endothelialization using SEM and assessed for neointimal proliferation by histomorphometry.

### Interventional procedure

All animals were pre-treated with a daily dose of 325 mg acetylsalicylic acid and 75 mg clopidogrel for 3 days prior to surgery and on each subsequent day until death. Twenty- seven domestic juvenile pigs, 30–35 kg in weight, were implanted with an SES, GS or HCASES in the left anterior descending and left coronary artery or right coronary artery. Sedation was achieved with intramuscular injection of ketamine (25 mg/kg body weight), xylazine (3 mg/kg body weight), and acepromazine (0.2 mg). Surgical access was obtained by cutting down on the left femoral artery. An intravenous bolus of 5000 IU of heparin was administered to achieve an activated clotting time of 250 to 300 s during the procedure. Before placing catheters to perform baseline angiograms, nitroglycerin was administered intra-arterially to relieve vasospasm. According to angiographic analysis of the vessel size, the appropriate stent was delivered to the intended site via fluoroscopic guidance, and stent implantation was performed (stent-to-artery ratio of 1.1:1.0). Angiograms were performed immediately. After deployment, the catheters and introducer sheath were removed, the surgical incision was repaired, and the skin was closed in two layers. Animals were euthanized (at the designated time) by an intravenous injection of a lethal dose of sodium pentobarbital.

### Histological analysis

Hearts were excised and the stented segments, as well as adjacent vessels (>5 mm of the native vessel on both sides of the stent), were dissected from the heart. The treated arteries were pressure-perfused with 500 ml of 0.9% saline to wash away blood cells, and then they underwent perfusion fixation for 30 min at 100 mm Hg pressure with formaldehyde buffered in PBS (pH 7.4). The dissected vessel was cut transversely into two equal parts. The first segment was fixed in 2% glutaraldehyde for SEM, and the remainder was stored in 10% neutral buffered-formalin overnight for histological analysis. The arteries for histological sections were processed for epoxy embedding [13,14].

### SEM analysis

Arterial segments for SEM were sheared longitudinally to unmask the lumen surface and then dehydrated in a graded ethanol series. After critical point drying in carbon dioxide, the samples were mounted on specific stubs and sputter-coated with gold. The tissue specimens were observed using a JEOL JSM-T300 SEM (Tokyo, Japan). To present the entire appearance, low power photographs of 15×, 35× and 50× were taken of the luminal stent surface. Regions of interest were photographed at further magnifications of 200× and 500× for direct visualization of endothelial cells (ECs). The degree of endothelialization above and between stent struts was surveyed via morphometry software (Image-Pro Plus 6.0, Media Cybernetics, USA), and then reported as the percentage of endothelial coverage.

### Histomorphometric and histopathological evaluation

For histology, the dissected arterial segments were gently flushed with PBS for 30 s and placed in a 10% formalin/PBS solution for further processing. The sections were stained with hematoxylin & eosin (HE). Injury and inflammatory scores were assessed by a blinded pathologist according to the published methods of Schwartz (0–3) and Kornowski (0–3), respectively [13]. In addition, the cross-sectional areas (including external elastic lamina, internal elastic lamina and lumen) of each section were measured with digital morphometry. Areas of the stent and lumen were measured and the percentage of stenosis was calculated ([stent area - lumen area]/stent area ×100%). Neointimal thickness was defined as the distance from the inner surface of each stent strut to the luminal border.

### Statistical analysis

Continuous data are expressed as mean ± standard deviation. All data were analyzed using SPSS software (SPSS 13.0; SPSS, Chicago, IL). A comparison of continuous variables between groups (including luminal areas, neointimal areas, and neointimal thicknesses) was performed using one-way ANOVA analysis.

For endothelialization rates, statistical comparisons were performed using the Wilcoxon test for two groups or the Kruskal-Wallis test for three or more groups. A p value of ≤0.05 was considered statistically significant.

## Results

### Evaluation of stent endothelialization by SEM

**Figure 2:**
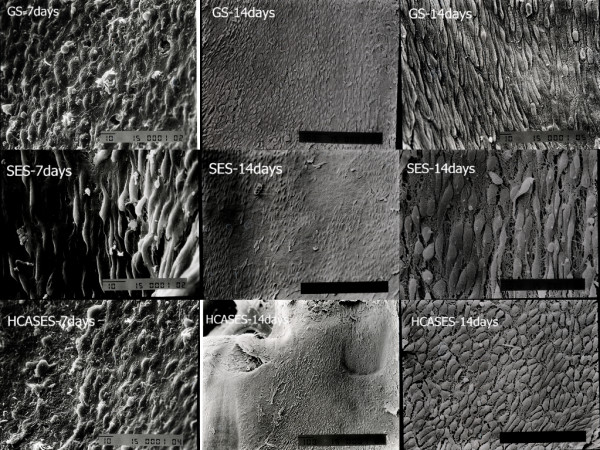
**Representative SEM images of the three types of stents at 1-week and 2-week follow-ups. **SEM (magnification×200) at 7 days showed the greatest endothelialization rates in the GS group ( GS-7days ), and there was also a significant increase in endothelial strut coverage in the HCASES group ( HCASES-7 days) compared with the SES group (SES-7 days). At 14 days, Low-magnification SEM images (50×) showed thin endothelial coverage on the stent struts of the three different types , and regions of interest were photographed at further magnifications of 500× for direct visualization of ECs.

**Figure 3:**
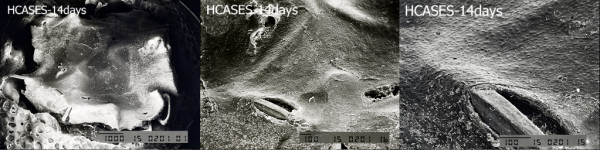
Representative lower-power SEM images of 15× and 35× for the whole face of the HCASES at 14 days.

A total of 18 stents, including six GSs, six SESs, and six HCASESs, were harvested at each time point. The ratio of endothelial coverage above the stents was evaluated by SEM at each time point. SEM analysis at 7 days showed the greatest endothelialization rates in the GS group (83±5%), and there was also a significant increase in endothelial strut coverage in the HCASES group (68±7%) compared with the SES group (31±4%; p=0.02; Table 1). High-magnification SEM images (200× and 500×) showed that GSs and HCASESs were closely covered with cells with an endothelial-like phenotype, while this was not the case with SESs. At 14 days, stent surface endothelialization evaluated by SEM showed a significantly higher extent of endothelial coverage above struts in the GS (95 ± 2%) and HCASES groups (87±4%) compared with that in the SES group (51±6%; p=0.02; Table 1). Low-magnification SEM images (15×, 35×, and 50×) showed thin endothelial coverage on the stent struts of the three different types (Figures 2, 3). However, high-magnification SEM images ( 200× and 500×) showed that endothelial strut coverage on SESs was partial and discontinuous (Figure 2). Stent surface endothelial coverage was nearly complete in the HCASES group (95±4%), similar to that in the GS group (97±3%) at 28 days, whereas the SES group (74±8%) showed the lowest endothelialization rates (Table 1). There was no difference in the rate of endothelial coverage between the HCASES and GS groups. Furthermore, high-magnification SEM showed that endothelial cells exhibited a cobblestone-like phenotype, indicative of a mature functional endothelium in the HCASES and GS groups.

**Table 1:** Quantitative Scanning Electron Microscopy:Percentage Endothelium Covering The Stent Struts at 7 Days, 14 Days and 28 Days

**Endothelialization (SEM)**	**GS**	**SES**	**HCASES**	***P *value**
**7 days (n=9 each)**				
Above struts	83±5%^§^	31±4%^*^	68±7%^§*^	0.027
Between	90±2%	74±8%^*^	85±5%^*^	0.044
**14 days (n=9 each)**				
Above struts	95±2%	51±6%	87±4%	0.022
Between	98±2%	89±4%^*^	95±3%^*^	0.031
**28 days (n=9 each)**				
Above struts	97±3%	74±8%^*^	95±4%^*^	0.042
Between	100±0%	94±2%	100±0%	0.079

^§^*P*<0.05 for GS vs HCASES.

^¶^*P*<0.05 for GS vs SES.

^*^P<0.05 for SES vs HCASES.

### Evaluation of stent efficacy by histology

**Figure 4:**
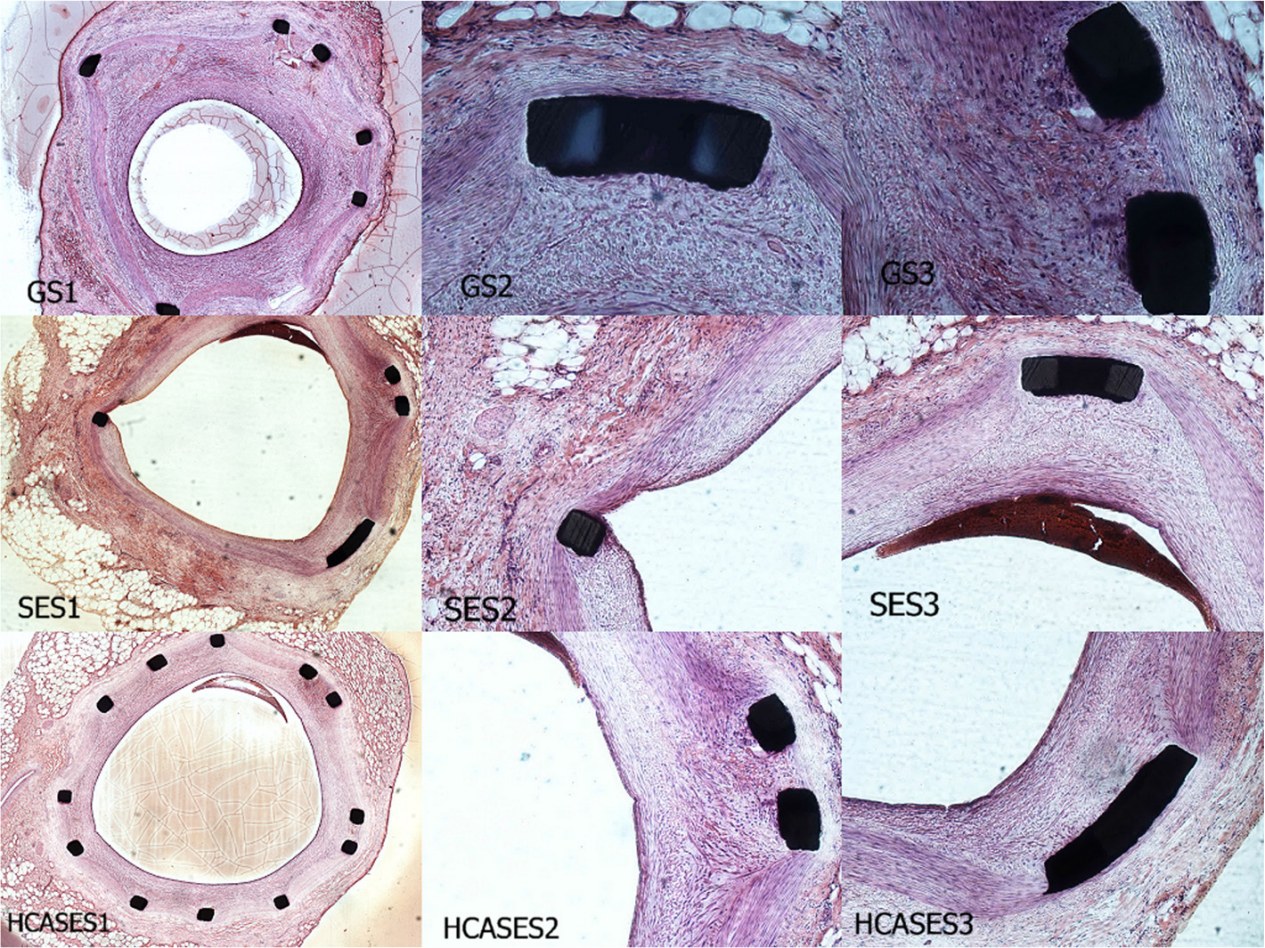
**Representative low-power photomicrographs (magnification×4) and high-power photomicrographs (magnification ×10) at 28 days after implantation of GS, SES and HCASES in porcine coronary arteries. **The HCASES group showed less stenosis than that in the GS group,and there was no significant difference compared with the SES group . Of note , stent struts( shown in GS1 and SES1) may be deformed by excessive strength when the dissected vessel is cut transversely into two equal parts.

At the 2-week follow-up, histological examination showed that the injury and inflammation scores were not significantly different between the three groups (p =0.436 and 0.482, respectively) because the stents were carefully deployed with a stent to artery ratio of 1.1:1.0. Similarly, the percentage of stenosis of the HCASES group was not different from that of the SES and GS groups (both p > 0.05) (Table 2). At 28 days, the SES group had the least amount of neointimal thickness compared with the other groups. However, the HCASES group showed less stenosis than that in the GS group (p < 0.05), but there was no significant difference compared with the SES group (p=0.068). In addition, there was less inflammation in the HCASES and SES groups compared with that in the GS group (Table 2). Figure 4 shows representative images of histomorphometry for each stent type. SEM and histological analyses demonstrated that HCASESs promoted enhanced endothelialization, when it reducd neointimal formation and inflammation at 28 days.

**Table 2:** Histomorphometric and Histopathologic Finding at 14 Days and 28 Days Following Implantation of GS, SES, or HCASES in Porcine Coronary Arteries

**Histological results**	**GS**	**SES**	**HCASES**	***P *value**
**14 days(n=9 each)**				
Injury score	0.77±0.24	0.84±0.19	0.92±0.26	0.436
Inflammation score	1.64±0.38^§^	1.01±0.21	0.81±0.29^§^	0.482
IEL area(mm^2^)	4.11±0.46	3.93±0.82	3.99±0.36	0.085
Lumen area (mm^2^)	2.76±0.43	2.99±0.53	2.84±0.33	0.091
Intimal area(mm^2^)	1.82±0.94	1.34±0.49	1.58±0.66	0.058
Neointimal thickness(μm)	145.39±72.57	112.72±38.52	136.17±51.94	0.072
Percent of stenosis (%)	17.88±8.24	13.94±5.21	15.68±7.03	0.066
**28 days(n=9 each)**				
IEL area(mm^2^)	4.76±0.84	4.36±0.53	4.49±0.46	0.658
Lumen area (mm^2^)	1.96±0.47^§^	2.78±0.36^*^	2.55±0.62^§*^	0.037
Intimal area(mm^2^)	2.80±0.37^§^	1.58±0.17^*^	1.94±0.84^§*^	0.024
Neointimal thickness(μm)	276.25±108.36^§^	131.44±43.85^*^	184.53±87.29^§*^	0.022
Percent of stenosis (%)	33.74±11.85^§^	16.48±7.31^*^	20.51±9.43^§*^	0.031
Injury score	0.81±0.14	0.79±0.24	0.82±0.16	0.462
Inflammation score	1.69±0.33^§^	1.21±0.72	1.07±0.56^§^	0.447

Data are mean±SD. IEL, internal elastic lamina.

^*^P<0.05 for SES vs HCASES.

^§^*P*<0.05 for GS vs HCASES.

^¶^*P*<0.05 for GS vs SES.

GS = anti-CD34 antibody stent; SES= sirolimus-eluting stent.

HCASES = HC-anti-CD34 antibody combined with a sirolimus-eluting stent.

## Discussion

Despite the fact that the SES has greatly reduced the incidence of in-stent restenosis compared with bare metal stents, delayed re-endothelialization may increase the risk of late-stent thrombosis and high lethality [6,11]. It is imperative to hasten the healing of injured endothelium after stent implantation. In the current study, delayed re-endothelialization in SESs was ameliorated by a novel combination of coating of HC-anti-CD34 antibody, which was added to a cardiovascular device to accelerate endothelialization. A coating of anti-CD34 antibody has been reported to cause rapid re-endothelialization by the attraction and adhesion of circulating EPCs and has proven to be feasible and safe by recent studies [15,16]. Furthermore, HC has been shown to be non-toxic and non-anaphylactic, with an excellent biocompatibility in vivo, which contributes to promote the establishment of healthy, functional ECs [12,17]. In our study, both histological and SEM examination demonstrated that the coverage rate of stent struts in HCASESs (Above struts: 87±4%, Between struts: 95±3%, 14 days) was to be well matched with that in the SES–anti-CD34 stent (Above struts: 82±8%, Between struts: 96±2%, 14 days) as previously reported [10]. These results indicated that the combination of a coating of HC-anti-CD34 antibody applied on SESs is more suitable for the enhanced adhesion of EPCs and growth of ECs than EPCs captured on SESs alone. Min Yin et al. reported a combination of coating with mussel adhesive polypeptide and anti-CD34 antibody on the surface of vascular stents, which can significantly enhance the attachment of EPCs as well as ECs, and greatly reduce platelet adhesion, indicative of better blood compatibility [18].

Recently, an increasing amount of researchers have investigated endothelial function and maturity (CD31 expression) [4,5,19]. Nakazawa et al. used confocal microscopy to assess the presence of mature ECs by the detection of CD31/PECAM-1- positive cells [10]. However, accumulating data have shown that there is a disparity between endothelialization rates by SEM and PECAM-1 positive rates by confocal microscopy analysis [10,20]. We consider that this disparity may result from dysfunctional ECs without PECAM-1 expression. Endothelial dysfunction may be related to the nonselective effect of inhibition of sirolimus on ECs and EPCs. Furthermore, in vitro cell culture studies have demonstrated that the antiproliferative concentration (−log IC50:5.23 ±0.08) in smooth muscle cells is similar in ECs (4.80 ±0.05) and EPCs (5.14 ±0.03) [10,21,22]. In the current study, HC coating promoted the adhesion of EPCs and the growth of ECs, and then the synergistic effect of HC-anti-CD34 antibody coating offset the inhibitory effect of sirolimus on ECs as well as EPCs. However, a large percentage of endothelial coverage by SEM does not equate to healthy endothelial cells. Healthy and mature ECs may express eNOS, vWF, and CD31/PECAM-1 [10,23]. Although we could not assess the maturation of the endothelium or ECs (eNOS, vWF or CD31/PECAM-1 expression) via immunocytochemistry or confocal microscopy, the rate of endothelialization was increased by 45% by 28 days in the HCASES, which was similar to that in the GS (up to 97±3%). Therefore, the combination coating of HC-anti-CD34 antibody enhanced stent endothelialization by offsetting the inhibitory effects of sirolimus on ECs and EPCs when applied on SESs. The major challenge in the present study was to confer the potential benefit of HC-anti-CD34 coating to a stent system in vivo.

Our results showed the short-term (4-week follow-up) protective effect of HCASESs on neointimal hyperplasia. Histopathological evaluation confirmed that HCASESs displayed a lower amount of neointimal hyperplasia and less inflammatory reaction compared with GSs, while maintaining EPC recruiting potential via HC-anti-CD34 coating. These results are consistent with the notion that sirolimus is characterized by immunosuppressive and anti-inflammatory effects [18,24-26]. Nevertheless, it has been reported that SESs might lead to the formation of atherosclerotic and thrombogenic neointima in the stent-implanted site [27,28]. This phenomenon may be concerned with endothelial dysfunction after SES implantation. Moreover, there has been considerable debate on the long-term safety and efficacy of SES, given the potential for late stent thrombosis, as well as possibly late catch-up in restenosis. In a sense, we think SES might be considered obsolete because of its potential risk of late stent thrombosis. Accordingly, further studies are required to determine the potential mechanisms of in-stent restenosis and vascular healing in the condition of atherosclerosis.

## Conclusion

Our findings indicate that the combination coating of HC- anti-CD34 antibody applied on a SES (HCASES) can promote endothelialization, while reducing neointimal formation and inflammation.

## Abbreviations

SES: Sirolimus-eluting stent; EPCs: Endothelial progenitor cells; HCASES: Hyaluronan-chitosan (HC) and anti-CD34 antibody applied on a SES; GS: Anti-CD34 antibody stent; SEM: Scanning electron microscopy; ECs: Endothelial cells; LST: Late in-stent thrombosis.

## Competing interests

The authors declare that they have no competing interests.

## Authors' contributions

BF, S-c F and FY designed the study, participated in the operation and performed the statistical analysis. S-c F drafted the manuscript. BF, FY and X-j P interpreted the data and revised the paper. S-x Z prepared the HCASESs. All authors read and approved the final manuscript submitted for publication.

## Pre-publication history

The pre-publication history for this paper can be accessed here:

http://www.biomedcentral.com/1471-2261/12/96/prepub
